# Communication barriers in aftercare: a qualitative study of allogeneic stem cell transplant patients

**DOI:** 10.1186/s12885-026-16274-x

**Published:** 2026-06-04

**Authors:** E. Wilhelm, G. Tavori, C. Bernardi, R. Ram, D. Wolff, A. Herrmann

**Affiliations:** 1 Department of Medicine III, University Hospital Regensburg, University of Regensburg, Regensburg, Germany; 2 Hematology Department, Tel Aviv Medical Center, Tel Aviv, Israel; 3 Medical School, Tel Aviv University, Tel Aviv, Israel; 4 Department of Epidemiology and Preventive Medicine, Division of Medical Sociology, University of Regensburg, Regensburg, Germany; 5 Bavarian Center for Cancer Research (BZKF), Regensburg, Germany

**Keywords:** Allogeneic hematopoietic stem cell transplantation, Physician-patient-communication, Framework analysis, Unmet needs, Qualitative research

## Abstract

**Background:**

Effective communication between patients and healthcare providers is a crucial component in aftercare after allogeneic hematopoietic stem cell transplantation (alloHSCT). Despite significant advances, unmet physical and psychological needs due to communication failures persist.

**Objective:**

We performed a multicenter cross-sectional study assessing domains not communicated and underlying causes to identify and comprehend communication gaps in alloHSCT aftercare.

**Study design:**

Semi-structured in-depth interviews were conducted with patients after alloHSCT at the transplant centres Regensburg and Tel Aviv. Framework analysis was used to examine the qualitative data.

**Results:**

The study recruited 47 patients with a median age of 57 years. A number of communication barriers were identified including (i): being overwhelmed during consultation preventing receiving and conveying information which interfered with early self-detection of chronic graft-versus-host disease; (ii) discrepancy in labelling psychosocial symptom load (e.g., psychological distress in part caused by war (Israel), cognitive impairment, adherence) being frequently neither recognized nor addressed due to discordance on who is considered responsible for bringing-up symptoms and issues considered as taboos, such as sexual health (iii) non-functioning chains of communication leading to loss of information. Other themes included strategies to compensate for communication deficits, i.e. (iv): buffering information gaps through social network and nurses. Finally, guidance and suggestions for communication and aftercare from the patient’s view were provided.

**Conclusion:**

Our qualitative study highlights significant communication gaps experienced by patients undergoing alloHSCT. The findings advocate for better communication training for healthcare professionals, improvement of the informed consent process and the development of prompt sheets for patients and checklists for physicians.

**Supplementary Information:**

The online version contains supplementary material available at 10.1186/s12885-026-16274-x.

## Introduction

Patients after allogeneic hematopoietic stem cell transplantation (alloHSCT) require intensive lifelong follow-up due to frequent long-term complications (e.g. acute and chronic graft-versus-host disease (cGVHD), infections, secondary malignancies and other comorbidities) [1, 2]. Long-term complications affect all domains of daily life including multiple organ impairment, the need for complex pharmacotherapy, physical and psychological comorbidities, and socio-medical challenges [3–5]. Moreover, neurocognitive impairments may also impair quality of life and rehabilitation [6]. The sum of these problems frequently result in relevant problems not being communicated nor addressed by either the patient or the physician in routine practice (e.g., neurocognitive problems) [7, 8]. The reasons are complex and involve both the patient (e.g., neurocognitive limitations, considered as non-reversable or unrelated) and the physician (lack of time and experience and structure, not regarded as competent or responsible) [9]. In line with this, a study in young adult cancer survivors and their providers revealed that the patients’ main barrier to symptom communication is physicians’ lack of time while physicians named patients not bringing up their symptoms [10]. Not surprisingly, a Community Advisory Board (CAB) held in February 2023 with patients with cGVHD after alloHSCT revealed multiple problems that are neither addressed nor communicated, which could not be solely explained by lack of resources [11] and two recent reviews underlined the lack of adequate communication between patients and healthcare professionals as relevant cause [8, 12]. An additional factor for failed communication is the lack of choice of the medical care provider since transplant aftercare can be only provided by selected physicians experienced in transplantation who may not necessarily be trained in communication [13]. 

Communication deficits may not only impair aftercare but already start before transplantation during the informed consent (IC) process, which frequently is perceived as overwhelming, leading to a lack of recall, which increases over time and impairs the ability to participate in shared decision making [14]. Moreover, patients frequently report a lack of knowledge of the early symptoms of cGVHD and other late effects often leading to delayed diagnosis [11]. In consequence, a significant proportion of patients are diagnosed for cGVHD already having severe symptoms which developed over weeks resulting in long term impairment of quality of life (QoL) and physical functioning (PF) [15]. In order to develop strategies to reduce communication deficits in follow-up care, we aimed to capture relevant domains, which are not addressed and to identify and comprehend underlying causes from the patients perspective applying a qualitative approach involving semi-structured patient interviews. The interviews were conducted at two sites located in different countries to permit an assessment independent of center effects. We purposefully selected Israel as second site for two reasons: first while still evolving a Western population the specific multicultural context of Israel may identify additional aspects using the same interview guide and second because of a specific interest of the Tel Aviv team to conduct a trial on communication gaps.

## Methods

### Study design

Patients after alloHSCT participated in a face-to-face semi-structured interview performed by an independent investigator not being part of the transplant team to exclude reporting bias. Inclusion criteria were an age *≥* 18 years, a follow up of at least 3 months after alloHSCT without upper limit, remission of the underlying malignancy, and German (Regensburg) or Hebrew (Tel Aviv) speaking. Patients were contacted directly after the physician contact in the outpatient dept. by DW (Regensburg) or GT (Tel Aviv) and informed about the study. Interviews were subsequently performed before the next physician contact after written informed consent to prevent any influence of outpatient visits on interviews. The treating physicians were not informed at any time on the participation in the study. Moreover, patients were ensured on the confidentiality of the content of the interview. Recruitment was performed applying theoretical sampling to include a broad range of participants with regard to age, time after alloHSCT, gender and socioeconomic background (Table 1) [16]. While both center were comparable in number of transplantation (60–70/year), organisational structure with outpatient care solely provided by the center incl. additional counselling by experienced nurses while psychological care is provided on demand only, medical care differed with the center in Tel Aviv providing care by two experienced attendings while the Regensburg center provided medical care by changing fellows supervised by one attending (DW). Moreover, patient characteristics differed as shown in Table 1, including time since transplantation, educational level, diagnosis distribution, and cGVHD severity. The study was approved by both institutional review boards (Regensburg: 23-3436_2-101; Tel Aviv; TLV-0721-23). The interview guide is provided in the supplement and was identical for both study sides with backward-forward translation being performed to ensure consistency. The interview guide was based on a literature search and topics raised during the above mentioned CAB meeting on 28th of February with cGvHD patients participating across Europe [4, 11, 12]. Interviews were recorded for transcription. Participants had the option to pause or reschedule the interview at any time. All interviews were opened with a narrative question on general perceptions regarding physician-patient consultations, and the subsequent interview was structured using the interview guide to ensure comparability. The interview guide was modified after the pilot phase (*n* = 5) to increase in-depth answers on topics seemingly important. The modification mainly addressed the reluctance of patients to “complain” communication gaps by asking more specific questions on coverage of mood disorders or sexuality. The interview guide consisted of questions about topic areas such as physical challenges, clinical care regarding GVHD, fatigue, neurocognitive decline and sexuality. Other aspects involved psychological issues and social well-being, and all topics were discussed regarding how they were communicated by the patient or physician or if they remained unaddressed by either party. Further questions involved what participants expected from physicians and if they had suggestions to improve care. The recruitment of patients was terminated after no new insights emerged after analysing five consecutive interviews indicating saturation of the data. The interviews and subsequent analysis started first in Regensburg and subsequently patients were recruited in Tel Aviv. When the ongoing framework analysis in Tel Aviv showed almost identical results compared to Regensburg except the war aspect and better comprehension of the informed consent indicating saturation of the sample recruitment was terminated resulting in a lower number of patients (*n* = 15) in Tel Aviv compared to Regensburg (*n* = 32).

**Table 1:** Patient demographics and clinical characteristics

Characteristics	No. of patients (%) Regensburg	No. of patients (%) Tel Aviv
Gender		
Female	15 (47%)	6 (40%)
Male	17 (53%)	9 (60%)
Age groups		
18–39 yrs	5 (16%)	0
40–59 yrs	12 (37.5%)	5 (33%)
Over 60yrs	15 (46.5%)	10 (66%)
Years since alloHSCT		
0–2 yrs	6 (18.8%)	12 (80%)
2–5 yrs	14 (43.8%)	3 (20%)
5-10yrs	6 (18.8%)	0
> 10yrs	6 (18.8%)	0
Occupation		
Full time	5 (15.6%)	4 (26.7%)
Part time	7 (21.9%)	5 (33.3%)
None/Pension	20 (62.5%)	6 (40%)
Highest form of education		
University degree	9 (28.1%)	11 (73.3%)
No university degree	23 (71.9%)	4 (26.7%)
Marital status		
Married	23 (71.9%)	11 (73.3%)
Stable relationship	5 (15.6%)	1 (6.7%)
Single/Widowed	4 (12.5%)	3 (20%)
Social situation		
Lives with family/friends	27 (84.4%)	11 (73.3%)
Lives alone	5 (15.6%)	4 (26.7%)
Children		
Yes	23 (71.9%)	14 (93.3%)
No	9 (28.1%)	1 (6.7%)
Underlying Diagnosis		
Acute myeloid leukemia	11 (34.4%)	11 (73.3%)
Acute lymphoblastic leukemia	2 (6.3%)	1 (6.7%)
Non-Hodgkin Lymphoma	6 (18.7%)	1 (6.7%)
Chronic myeloid leukemia	3 (9.4%)	0
Hodgkin Lymphoma	1 (3.1%)	1 (6.7%)
Myeloproliferative disease	5 (15.6%)	1 (6.7%)
Inborn immunodeficiency	1 (3.1%)	0
Aplastic anaemia	3 (9.4%)	0
cGVHD severity at time of the interview		
None	8 (25%)	6 (40%)
Mild	/	2 (13.3)
Moderate	2 (6.3%)	3 (20%)
Severe	22 (68.7%)	4 (26.7%)

Abbreviations: *yrs* years, *No* number

### Data analysis

The recorded transcripts were analyzed after verbatim [17] transcription using a whisper- based transcription tool (atrain and noscribe (Regensburg), turboscribe (Tel Aviv)). To ensure accuracy, EW and GT reviewed each transcript by listening to the audio recordings again and revised the transcripts accordingly. Consequently, a framework analysis was conducted separately for both sites [18]. First, an inductive approach was taken in which the transcribes were read intensively by EW and GT and coded openly by applying a paraphrase (i.e. a “code”). Codes were subsequently discussed with the supervisor (AH) with each cohort (Israel and German) being discussed separately. The codes described what had been identified as significant within the respective passage. Then, the codes were clustered into more complex categories through the summarization and synthesis of the coded data, thereby constructing an analytical framework. Multiple codes related to the same topic were used to form a category. The final framework was discussed again with AH for each site and then applied analyzing subsequent interviews. Following, all transcripts were coded by the two coders (EW and GT) using the analytical framework with independent coding of both cohorts (Regensburg and Tel Aviv) to ensure detection of potential differences. If new codes emerged, the framework was adapted. The dynamic analysis allowed for the continuous testing and refining of the hypotheses formulated with the help of the categories. After the categories were formed and interpreted, connections were established between them. This led to the next stage of the data analysis, which involved defining the main themes. Themes were considered concepts that describe and summarize core aspects within the dataset and served as the end result of the comprehensive analysis of the entire data gathered within this study. Finally, the data were analyzed and discussed by all members of the research team. Data management and analysis was conducted with the help of the ATLAS.ti (Tel Aviv) and MaxQDA (Regensburg) software [19]. 

## Results

Forty-seven patients were asked to participate, and all complied with the request (consent rate: 100%, response rate: 100%). The median time post alloHSCT was 3 years (range, 5 months – 26 years). Median duration of the interviews was 52 (range, 19–136) minutes. *Detailed patient characteristics are provided in Table* 1. The results revealed 5 different domains affected by communication deficits which can be divided into two categories as follows (i): Being overwhelmed during consultation as an obstacle for receiving and conveying information relevant in IC and shared decision making (SDM); (ii) Discrepancy in labelling of health issues relevant in communication on fatigue, psychological distress, cognitive dysfunction, sexuality; (iii) Non-functioning chains of information leading to loss of information relevant in structural aspects in aftercare. The second category consists of different approaches on how to reduce these deficits in communication, i.e. (iv): buffering gaps of information through social network; (v) guidance/suggestions for communication and aftercare from the patients view.

### Being overwhelmed during consultation as an obstacle for receiving and conveying information

Most of the respondents transplanted in Regenburg stated that they felt overwhelmed during the physician-patient consultations. This already applied to the pre-transplant informed consent session, with potential significant consequences for aftercare since long term consequences were hardly memorized. In particular, the flood of information, of which patients effectively memorised very little, led to gaps in knowledge about the disease and the course of therapy. A few patients mentioned that this also affected early recognition of cGVHD and in consequence they had trouble identifying it as such resulting in delayed diagnosis and treatment. Interestingly, being overwhelmed was less frequently reported by patients transplanted in Tel Aviv, taking into account that outpatient service was provided by two experienced attendings only trained in communication and in contrast to Regensburg most patients had an academic degree. Detailed patient quotes on the topic are provided in Table 2.

**Table 2:** Quotes from patients on being overwhelmed during consultations

Categories	Quotes from patients
Impact of consultation characteristics, procedure and content delivery	*So*,* during the counselling session back then*,* of course you get the explanation of everything that can happen afterwards. And that it might not work and*,* and*,* but then you just forget that again. (female patient*,* 50 years old*,* 8 years post-transplant*,* Regensburg)*
	*“They explained things to me during the hospitalization, and I also read the booklet, but it felt like too much information. For someone like me, detailed explanations were not helpful. I preferred to know things in general terms rather than being overwhelmed with details.” (female patient, 58 years old, 2 years post-transplant, Tel-Aviv).*
Anxiety due to the diagnosis and potential life-threatening consequences interfere with concentration	*If that doesn’t work*,* then you’ve lost. That’s the quintessence of the conversation. You’re just shocked. (male patient*,* 44 years*,* 1 year post-transplant*,* Regensburg)*
	*Too much information can be harmful. (male patient, 71 years old, 1 year post-transplantation, Tel Aviv)*
Reduced physical condition, neurocognitive impairment, psychosocial burdens or fatigue affected the ability to participate in discussions with their physicians with frustration about multiple failed therapies contributing	*I didn’t think that it’s so difficult to get under control. I thought that it’s something that goes away again quickly*,* not quickly*,* but it will pass again. But all the remedies we tried failed. (female patient*,* 67 years old*,* 4 years post-transplant*,* Regensburg)*
	*It’s always, the therapy doesn’t work again, then you do the next therapy, sometimes you’re just tired of the therapy. (female patient, 54 years old, 26 years post-transplant, Regensburg)*
	*It always depends on which doctor you have, some use a lot of technical terms, so you think, no idea. [.] was very quick and very fidgety and used a lot of technical terms. And I was glad that I still had my mum with me because I was sitting there and actually had far too much information. And, yes, we always guessed and pieced together afterwards what the doctor had said. (female patient, 26 years old, 1 year post-transplant, Regensburg)*
Perceived lack of health competence to participate in shared decision	*You can’t contribute because it’s the doctor who decides. You don’t know your way around. You have to trust them. (female patient*,* 50 years old*,* 8 years post-transplant*,* Regensburg)*
	*The way I see it, the doctor has his records and decides what he thinks is best for my health. And that’s what I do. […] I take it without discussion. Because it makes no sense at all to debate with a doctor. I don’t know my way around. I have to rely on the fact that it’s right. Because the doctor doesn’t do it for nothing. […] You can’t make any suggestions to the doctors. (male patient, 65 years old, 5 years post-transplant, Regensburg)*

Anxiety due to the diagnosis and potential life-threatening consequences and its associated treatment was often mentioned as stressor impairing to concentrate on the consultations. In addition, several participants stated that aftercare was at times frustrating because multiple lines of inefficient treatment of cGVHD including participation in studies did not provide satisfying results.

During physician-patient consultations, participants were unable to grasp the information either because of a different level of speech, including the use of professional jargon, the pace in which the knowledge was delivered, or the fact that the diseases and treatment are very complex in general. Noteworthy, patients repeatedly stated having slim to no competence to participate and discuss with their physicians about their therapies and medical decisions. Most reported that they had to trust their physician because as a layperson they thought not to possess enough knowledge about their condition and the treatments available to them.

All the abovementioned aspects added to the lack to participate in the decision process almost excluding SDM due to perceived power-imbalance and the numerous challenges patients face after alloHSCT.

### Discrepancy in labelling health issues

This study identified a discrepancy in labelling of health issues with no major differences between both sites (Regenburg and Tel Aviv). Some patients believed that for example genital GVHD was not discussed because it is thought to be a rare a complication. Others struggled with determining which symptoms were important enough to be discussed with the physician. Occasionally, patients expected no solution for their problem such as fatigue or neurocognition preventing them from sharing the symptom. Additionally, some patients linked symptoms such as physical weakness, issues with sexuality or neurocognitive deficits to their age instead to treatment, which prevented bringing up complaints (Table 3).

**Table 3:** Quotes from patients on discrepancy in labelling health issues

Categories	Quotes from patients
Differences in labelling health complaints in which patients expect no help from physicians or label complaints as unrelated or/and age associated	*No*,* [I did not discuss the fatigue with the doctor]*,* because I’ve had it for a long time and I can’t say I’m always tired every time I come*,* you have to do it yourself. (female patient*,* 50 years old*,* 7 years post-transplant*,* Regensburg)*
	*“My emotional state did not come up in the conversation, neither from the doctor’s side nor from mine. I didn’t feel the need to share it, because I felt that I was coping. I can’t really say whether it’s better or worse, but at the moment I’m managing. I have a caregiver, and that helps me a lot.” (female patient, 74 years old, 3 years post-transplant, Tel-Aviv)*
	*That wasn’t discussed at all. Not at all. But that is a problem. Because if you’re not doing so well, then you just don’t feel like it [= sexual intercourse]. And then you have a bit of a guilty conscience towards your partner, of course. That’s clear. [I didn’t mention it] because I thought that no one could help me. (female patient, 67 years old, 4 years post-transplant, Regensburg)*
Patients and physicians prioritize “medical” issues over psychosocial complaints	*The whole psychological thing was pushed to the back a bit*,* because then there were just so many other things to talk about. […] I sometimes had the feeling that decisions were not made based on me*,* but because there was some kind of template. Sometimes I had the feeling that it was a bit of a brush-off. (female patient*,* 29 years old*,* 5 years post-transplant*,* Regensburg)*
	*I’m just told, yes, but the lab values fit, the lab values fit. Yes, what use is it to me if the values fit if I feel so completely broken. […] You can’t just go after the lab values. (male patient, 59 years old, 4 years post-transplant, Regensburg)*
	*No, she didn´t ask about my emotional state (male patient, 73 years old, 2 years post-transplant, Tel Aviv)*
Symptoms labelled as new normal without discussing it	*No [I did not discuss it]*,* but I think it’s a burden for everyone*,* so anyone who says it’s not a burden is really crazy. (male patient*,* 71 years old*,* 2 years post-transplant*,* Regensburg)*
	*It [= sexuality] was not asked. It was said that sexuality is impaired. Before the transplant. That was later confirmed. (male patient, 55 years old, < 1 year post-transplant, Regensburg)*
	*There weren’t really severe symptoms… I was relatively fine. (male patient, 73 years old, 2 years post-transplant, Tel Aviv)*
Differences in perceptions of roles between patients and physicians	*I think every healthy person would realize for themselves if something [= genital] is wrong*,* then one would address it. I think some things are important for self-observation or simply for your own things*,* that you should actually see yourself. Honestly you can’t expect the doctor to ask everything in detail. (male patient*,* 55 years old*,* 6 years post-transplant*,* Regensburg)*
	*With a psychiatrist or a psychologist, I do share these emotional aspects. With the Professor, it felt less relevant. Not because of a conscious decision, but because it didn’t feel like his domain. In my view, there are professionals who deal with the emotional world, and he deals specifically with the transplant. That’s how I perceived it — that his role ends with the transplant.” (male patient, 42 years old, 1 year post-transplant, Tel-Aviv)*

Another key factor was the prioritization of other concerns, often related to the treatment itself. Many patients reported that psychosocial issues, sexual health, and fatigue were rarely communicated because these topics were not regarded as urgent compared to their immediate medical needs especially for patients suffering from GVHD. Participants were often preoccupied with the physical aspects of recovery and focused on the essential survival and cancer-related concerns. To some patients, it seemed that physicians also ranked peripheral issues lower as they focused more on haematologic burdens, laboratory results and going through their distinct approach during consultation leaving less room for seemingly “smaller” issues. This ranking was supported by the assumption that a significant number of symptoms were recognized as “normal” after alloHSCT. Being tired, physically exhausted, anxious or emotionally distressed was expected as part of recovery and may resolve by time which contributed to a reluctance to communicate issues that might otherwise have warranted attention.

An additional reason for the lack of communication was the differing perceptions of roles between patients and physicians. Many patients felt that they were responsible for addressing their own concerns, particularly when it came to “secondary” issues like sexuality or psychosocial well-being. Participants also argued that they had to keep an overview of their medical history, shared responsibility in overseeing the medication and had to work through social matters or psychological burdens on their own. This role of self-advocacy was often driven by the belief that the same topics were not part of the physician´s job description.

### Non-functioning information chains leaving problems unsolved

The repetitive replacement of physicians in Regensburg was perceived as disruptive, with the majority of patients feeling a lack of familiarity and emotional connection with new providers making it difficult to open up. This disruption hindered the holistic understanding of the patient’s condition, and the information transfer was reversed as patients had to reshare their complex medical history. It was noted that having a reference physician at the start of aftercare and during treatment of cGVHD for a longer period could improve care and help with adjusting while it was perceived less crucial for long-term standard aftercare. Additionally, a few patients from Regensburg indicated that in case of emergencies at night or weekend, physicians being on call did not know their medical history and patients felt these unknown providers should take more precautionary measures in case of complications. In contrast, at the Tel Aviv transplant center attendings from the alloHSCT team only provide on call service for alloHSCT patients. A small number of participants at the Regensburg center welcomed the occasional change of physicians, for their new perspective including improved treatment.

A repetitive additional complaint was lack of interdisciplinary exchange which is an essential component of multidisciplinary care in alloHSCT survivors including various disciplines such as dermatology, ophthalmology, and gynaecology resulting in either ignorance of problems or conflicting advice. Even worse were experiences with medical care outside the transplant center lacking expert knowledge resulting in misdiagnosis and wrong treatment in the context of cGVHD.

Almost all patients reported organizational challenges including long waiting times, delays in laboratory results, and high patient volumes mainly in other clinics. These factors occasionally led to reduced willingness to ask questions or communicate concerns during consultations. Few patients reported that their psychological care was significantly impacted by long waiting times outside the clinic infrastructure, leaving patients with insufficient opportunities for addressing mental health concerns.

The perception of non-verbal communication contributed to a failure in sharing important health information. Some patients mentioned that they were probably not asked about certain topics e.g. psychological distress because of their nature or appearance. Some topics were not addressed by the patients for example physical weakness because the physician would have noticed them anyway. Other instances included patients being queried about their condition via questionnaires which were not discussed with the physician leading to unprocessed information. A significant proportion of patients reported that private or “taboo” issues, especially related to sexuality, were not discussed, with gender specific barriers or the presence of a third person (nurse) not solely explaining the lack of communication.

Support persons were often a valuable source of support and in select cases had a better overview of the patient’s condition, particularly regarding oral medication and implementation of treatment requirements especially shortly after alloHSCT. In line, the absence of support persons during consultations sometimes resulted in lack of understanding of treatment details by patients as patients were sometimes overwhelmed and needed someone else to ask essential questions and later to discuss the influx of information. In rare instances, advice from caregivers was followed without consulting the physician, leading to discontinuation of medication due to side effects without professional input (Table 4).

**Table 4:** Quotes from patients on non-functioning information chains

Categories	Quotes from patients
Deficits in physician patient relationship due to replacement of the physician	*In my opinion*,* if there is frequent change*,* the doctor who comes in for the first time doesn’t even know what’s actually going on with me. He only sees numbers and data. (male patient*,* 68 years old*,* 7 years post-transplant*,* Regensburg)*
	*It’s a bit like starting from scratch, explaining a bit, these are my symptoms, and no, I don’t need this medication, and no, I don’t want to stop taking it. And that you always have to start from the beginning with that person. And of course, you also build up a bond with those you already know. And then, you naturally lack the confidence to address certain things yourself. (female patient, 29 years old, 5 years post-transplant, Regensburg)*
	*We can write to him on WhatsApp… and get immediate guidance.” (female patient, 63 years old, 3 years post-transplant, Tel Aviv)*
lack of interdisciplinary exchange	*I was in [a rural] hospital and the gastroenterologist didn’t send the sections to [a bigger city]. And that was another thing where you say that the pathologist in a hospital with a population of 70*,*000 may not be able to tell whether it’s GvHD or not because he doesn’t have any experience with it.[…] When everyone works together a bit*,* I have a better feeling*,* because I know from my experience that*,* for example*,* in case I need antibiotics or I don’t need antibiotics*,* the BMT usually assesses things differently than the general practitioner*,* so I always have a bad feeling when a doctor says*,* no*,* no*,* I don’t need to contact them. (male patient*,* 48 years old*,* 20 years post-transplant*,* Regensburg)*
Structural deficiencies like long waiting times and lack of access	*Of course*,* if you’re just sitting there*,* awake and waiting for the results to come in and goodbye. Of course*,* you’re tempted to do that*,* because I’ve been there since 7:45*,* sitting around the whole time*,* and of course everyone is groggy. I’m glad when I’m out again. […] If you’re there for 4–5 h and you go to the doctor’s appointment*,* you just want your documents. Is everything ok*,* then goodbye*,* you just want to go home. You don’t feel like talking about every detail anymore. And then when you get home*,* you notice something that you should have discussed. (male patient*,* 65 years old*,* 5 years post-transplant*,* Regensburg)*
	*It’s not a closed room… behind a curtain.” (female patient, 63 years old, 3 years post-transplant, Tel Aviv))*
False assumptions due to non-verbal communication	*I think that [= emotional distress] is also something they notice when you walk into the doctor’s office*,* I guess. So far no one has asked me about it. (female patient*,* 69 years old*,* 1 year post- transplant*,* Regensburg)*
	*And I think the doctors have noticed that [I can handle anxiety]. At every appointment, I have a note with me where I’ve thought about what I want to say the day before, so that had and has structure. And they already recognized that I wasn’t talking wildly. I mean, they already understood from their experience that it’s not a patient who’s suffering from depression. (male patient, 56 years old, 5 years post-transplant, Regensburg)*
Essential role of support persons	*At home*,* my wife was there for me and helped me a lot. She also made sure that my diet and medication were correct. (male patient*,* 68 years old*,* 7 years post-transplant*,* Regensburg)*
	*My partner manages everything… I don’t really speak, he speaks.” (female patient, 63 years old, 3years post-transplant, Tel Aviv)*
	*I don’t remember it because I don’t have to remember it. Here we go again, it’s another one of those feel-good things, I don’t have to remember it. That’s what my wife does. Maybe it’s not right, but that’s just the way it is. (male patient, 58 years old, 2 years post-transplant, Regensburg)*
Sexual health addressed only within compartmentalized care	*“Side effects related to sexual functioning or sexuality? Not really. Sexual desire is almost non-existent*,* I would say. And whether this came up with the Professor—no. It came up with we female attending*,* because she performed the gynecological examinations*,* but it was not a topic we went into in depth at all.”.” (female patient*,* 64 years old*,* 3 years post-transplant*,* Tel-Aviv).*

### Buffering gaps of information through the social network

Confiding in family members or friends often included sexuality as a more private theme and as such for some patients this was the preferred option at both sites (Regensburg and Tel Aviv) analysed. Other areas were psychological issues such as anxiety, emotional distress as well as social problems e.g. financial issues, becoming accustomed to a new social role or isolation due to the risk of infection.

If discussed with the medical team, these topics were mainly conversed with nurses (link-nurses) working in the outpatient department. These nurses knew the patients during inpatient treatment already and were afterwards available for the transition to outpatient life and stayed available long-term. Almost all patients identified these nurses as essential for addressing informational gaps ranging from medication, nutrition to help with social matters and were sometimes preferred because of the emotional connection.

Lots of gaps were closed by the patients themselves as they often tried to solve issues on their own by either searching the internet, reading through package leaflets concerning medication side effects or in some cases by talking with other survivors. It must be mentioned that a few patients did not welcome the idea of conversing with other patients as they felt that “only bad things are discussed” (female patient, 50 years old, 9 years post-transplant) and preferred to contact their physician or medical team, if they did not find a solution by themselves. In some instances, topics remained unaddressed because patients did not want to burden the team social or psychological issues because of the perceived too high workload of physicians (Table 5).

**Table 5:** Buffering gaps of information

Categories	Quotes from patients
Role of family member and friends	*Yes*,* [I would not talk to the doctor about sexuality]*,* that’s my problem. […] I’ll sort it out with my husband. (female patient*,* 67 years old*,* 2 years post-transplant*,* Regensburg)*
Role of liaison nurses	*The link nurses were very good. It helped me a lot. Not just me*,* but also my family. Some of my family also took part in conversations so that we knew how things would work at home. (male patient*,* 34 years old*,* 7 years post-transplant*,* Regensburg)*
	*The link nurses were excellent with the disabled person’s pass, with all the stuff we needed. […] you have no idea what [the social matters are] about. That’s where the help comes in. […] Without link nurses, you’re often lost. (male patient, 44 years old, 1 year post-transplant, Regensburg)*
Fear to become a burden to the staff	*I would say that if something new occurs*,* I would always have a look myself first to see what it could be […]*,* discuss it with my mom beforehand or ask Dr. Google. (female patient*,* 28 years old*,* 8 years post-transplant*,* Regensburg)*
	*Not that I don’t trust the doctor*,* but first*,* before I ask a stupid question*,* I would try to find information myself about what and how. (female patient*,* 54 years old*,* 2 years post-transplant*,* Regensburg)I don’t want to disturb them because I know how busy they are. (female patient*,* 54 years old*,* 5 years post-transplant*,* Regensburg)*
Shift of illness management from patient to caregiver	*“To be honest*,* my partner manages my illness. If you asked him*,* he would be the expert. He manages all the medications to this day. They always joke that when we come to appointments*,* I don’t speak—he does. We keep Excel spreadsheets and write down every day what I take and at what time.” (female patient*,* 64 years old*,* 3 years post-transplant*,* Tel-Aviv).*

### Impact of war

Since the interviews were performed during the war in Israel the latter had significant impact on the perception of patients. Besides disrupted or lost family support, uncertainties on housing after discharge and challenges in outpatient access to medical care, the situation of an armed conflict significantly framed prioritization ranking safety of family members and surviving the war higher than other highly relevant medical and psychosocial concerns thereby significantly contributing to communication gaps (Table 6).

**Table 6:** Impact of war

Categories	Quotes from a patient
Impact of war	*It was me and my partner. My children were also evacuated — actually*,* they were relatively close to me*,* by coincidence*,* because they were all around Tel Aviv — but the rest of the family was relocated to another town. So*,* the whole family was there*,* and I was going through the transplant in Tel Aviv*,* coping with everything*,* while having a kidnapped nephew — with all the worry*,* which I can’t even begin to describe — and then*,* eventually*,* the news of his death.* *I always say — and it’s not pleasant to admit — that having two fronts*,* two “wars*,*” so to speak*,* at the same time*,* somehow made it a bit easier for me. It’s not nice to say… but I admit I was more focused on my nephew’s situation and on the family than on my illness.” (female patient*,* 64 years old*,* 18 months post-transplant*,* Tel Aviv)*
	*Having two ‘wars’ at the same time.” (female patient, 63 years old, 3 years post-transplant, Tel Aviv)*

### Guidance/suggestions for communication and aftercare from the patients view

Almost all patients emphasized the need for a friendly and open environment during outpatient visits. They reported that familiarity and trust with the treatment team significantly improved their overall experience. Patients also highlighted the importance of not transferring stress to them during consultations. Furthermore, professional yet approachable staff who radiate calmness and demonstrate empathy was deemed helpful by patients during their recovery.

Most patients preferred clear, structured conversations where they could ask questions freely without feeling rushed. They valued opportunities for repeated inquiries and appreciated when staff took the time to ensure patients felt recognised. Questions posed in an active and engaging manner helped them feel involved in their care and was considered essential for addressing certain topics such as fatigue, sexuality or psychosocial issues. Using lay language, and providing direct, honest answers were regarded vital for creating a relaxed, open environment that encouraged patient participation.

Patients also expressed the need for sensitive and respectful interactions, where their concerns were taken seriously without stereotyping or a power imbalance. They valued healthcare professionals who established an emotional connection and possessed positive characteristics such as reassurance, empathy, and sympathy. These were mentioned as key elements in building trust and feeling understood and supported. A small number of patients called for thinking outside the box and giving more importance to “peripheral issues” i.e. psychosocial issues, secondary illness or sexuality.

From an organizational perspective, patients expressed a desire for better coordination between various healthcare providers, including specialists and general practitioners. They welcomed clear communication during emergencies and requested timely referrals concerning issues like cGVHD management, psychological concerns, or social challenges. Patients with longstanding experience or medical backgrounds felt more comfortable as active participants in their care, often seeing their physician as a consultant/partner. This sense of involvement allowed them to ask more questions, further enhancing their understanding and experience in a whole which calls for the general significance of patient empowerment (Table 7).

**Table 7:** Suggestions for communication from patients

Categories	Quotes from patients
Open and friendly environment and clear structure of conversation	*Because I say it in my own words and the doctor can explain it to me in a way that I can understand. Not with technical terms or medical jargon and stuff like that. The doctor speaks so well that I understand. (male patient*,* 64 years old*,* < 1 year post-transplant*,* Regensburg)*
	*I think if I was asked about it [= sexuality] or if it was put in the room like that, I would talk about it. (female patient, 29 years old, 5 years post-transplant, Regensburg)*
	*You can talk more easily to some doctors and sometimes, as I’ve already said, you feel a bit ignored, that it’s a done deal. And maybe, or of course, you can still say something, but you don’t have the feeling that it’s wanted. (male patient, 28 years old, 3 years post-transplant, Regensburg)*
Need for patient empowerment	*You grow into the whole thing over time*,* and you really become your own doctor*,* and you say*,* I’ll take care of myself*,* I know what’s normal*,* what’s not normal*,* because the doctors only see you once in a while*,* and so you have to listen to yourself. (female patient*,* 32 years old*,* 15 years post-transplant*,* Regensburg)*
	*If I have the feeling that a doctor doesn’t take me seriously, then I change doctors. […] But I have never perceived a doctor as an authority, but always as a kind of consultant who works with me. But I was always aware that I had to make the final decision. Just like I decided to have the transplant. This means that if I’ve been properly informed, I have to live with the consequences somewhere. (male patient, 48 years old, 20 years post-transplant, Regensburg)*

## Discussion

Patients undergoing alloHSCT often have unmet needs during follow-up care despite seeing their healthcare professionals [20–22]. Many patients from the Regensburg cohort shared that they felt overwhelmed during their consultations, especially during the pre-transplant IC. The sheer volume of information led to gaps in understanding and resulting in difficulties retaining key details about their condition and treatment. Anxiety about their diagnosis and the treatment process made it even harder to focus underlining the need to separate informed consent on alloHSCT from conversations on prognosis [12, 23, 24]. One option to increase recall is to provide 2 shorter consultations instead of 1 longer one to evade the feeling of being overwhelmed by reducing information influx [14, 25]. Cancer patients also preferred receiving both online and written information in relation to treatment options [14, 25]. Even though patients were informed about the challenges they might face after the transplant, they were often unprepared for the physical, cognitive, and emotional struggles that came with it. Participants wanted more education about post-alloHSCT quality of life, especially concerning late complications, which were often unexpected and impacted their sense of recovery (e.g., neuropathy, fatigue, cGVHD, osteonecrosis). Repetitive information at different times during aftercare about long-term side effects, their severity, duration, and impact on their lives may be helpful to increase awareness and permit adequate response [11, 26–28]. The IC process should include patients preferences and be tailored to their values [23]. Communication during consultations was another challenge. Some patients found it difficult to follow the conversation due to medical jargon, which should be provided in lay language [27]. Other barriers were the pace and complexity at which information was delivered. Alexander et al. found that in less than every third consultation, oncologists backchecked patients’ understanding of the information and cross check is highly recommended with Siminoff et al. highlighting the role of family members as additional source of comprehension and Barrata at al. adding the specific challenges of paediatric and adolescent patients in whom family member are an integral part of IC [21, 29, 30]. Galvano et al. drafted an approach for effective communication including simplifying the knowledge without altering the information and more guidance on how to prevent misunderstandings for communicating scientific knowledge in the cancer community [31]. The positive impact of physician education is underlined by the finding, that being overwhelmed during the IC was less frequently reported in the Tel Aviv cohort in which the two physician providing care received training in patient communication. Another factor which may have contributed to the finding that patients in Tel Aviv did not report the perception of being overwhelmed may be the significant higher percentage of patients with an academic degree which has been previously identified as predictor for better health literary in cancer patients [32]. 

The lack of sufficient information during the IC and aftercare resulted in multiple problems not communicated nor addressed. For example, patients perceived certain issues like genital cGvHD as too rare to discuss, despite the fact that sexual dysfunction is one of the most prevalent long-term side effects after alloHSCT [12, 33]. Others struggled with determining which symptoms were important enough to raise, particularly when they anticipated no solution, such as fatigue or neurocognitive problems [34]. Another study cited barriers such as time constraints, not knowing who to ask about fatigue, being regarded as weak and that physicians did not handle questions about fatigue well [35]. Additionally, patients tended to prioritize treatment-related issues, such as managing cGVHD, over psychosocial topics like sexual health or emotional distress. These were seen as less urgent, especially in the context of survival and immediate physical recovery [36]. Patients suffering from cGVHD often had to go through countless consultations, receive multiple treatments and medication and sometimes were tired of the inefficient therapies [11]. Thus, these patients need more encouragement to ask questions surrounding other topics as they are already overwhelmed dealing with cGVHD burdens and need more tailored care and individualised information transfer [4, 28]. The finding of prioritizing of physical symptoms in the presence of cGVHD over “peripheral issues” like psychological distress and fatigue is concerning since cGVHD is the dominant risk factor for these conditions which calls for systemic screening in clinical routine [37, 38]. 

Many symptoms, such as fatigue and emotional distress, were perceived as “normal” after transplant, which led patients to dismiss them as temporary and not worth discussing. Unmet needs such as fatigue and sexual problems are evident, but also do not seem to decline with more time post-transplant [39, 40]. One study found that oncologists believed sexuality to be an important part of their patients’ life but did not include it in their clinical care which aligns with the findings of the majority of patients not being asked about sexual related health problems despite the expectation of patients being actively inquired [11, 41] partially because healthcare professionals avoid discussing sexual concerns due to societal taboos, fear of embarrassing patients, and organisational barriers like time constraints and uncertainties about their role in addressing these issues [42]. Moreover, patients often felt responsible for addressing their own concerns, particularly regarding psychosocial well-being and sexuality. This sense of self-advocacy arose from the belief that these issues were outside the scope of the physicians role [43]. Furthermore, social or psychological distress remained unaddressed as patients sometimes hesitate to burden healthcare providers with “non-medical” concerns which calls for a more integrated psychological support in post-transplant care including application of screening tools which also foster discussion of intimate topics [36, 44–50]. In our study, patients complaint that questionnaires that they filled out before consultation were ignored which contributed to communication gaps although in general the use of questionnaires can lead to increased question asking and better patient satisfaction [49, 51, 52]. 

The crucial role of family or social network members as well as liaison nurses for informational support has been repetitively reported and confirmed by our study incl. patients after alloHSCT [27, 53–56]. The role of family members or other support persons is regarded essential during emotionally and physically challenging treatments incl. cGVHD [34, 57]. The absence of a social network even predicts poorer quality of life and a higher number of transplant complications [58]. This support can result in a significant distress on care providers [54, 59] and caregivers are regarded as an invisible part of the care team which in turn requires attention as well [60]. Despite these support systems, many patients sought to fill gaps independently, often by searching online or consulting other survivors [61]. While the internet offers accessible information and in some cases facilitates decision-making [62], it can also lead to non-adherence or misinformation [63]. eHealth-facilitated integrated care models (eICMs) although not applied in both cohorts have the chance to buffer gaps in aftercare and improve outcomes such as rehospitalisation [64–68]. 

This study underscores the importance of creating a supportive, open, and empathetic environment in alloHSCT care. Patients reported that familiarity with their treatment team, marked by calmness, empathy, and professionalism including structured communication, significantly improved their experience and sense of security, with patients appreciating the opportunity to ask questions without feeling rushed and revisit concerns as needed [13, 43, 69, 70]. calling for training of physicians in communication skills [71, 72]. From an organizational standpoint, patients called for better coordination between healthcare providers incl. access to organ specialists calling for a structured multidisciplinary care due to their special post-transplant needs which includes continuity of care [8, 73–76]. Patients greatly valued professionals who were aware of the boundaries of their own expertise and were not hesitant to seek advice from a colleague or consult scientific literature [8]. 

Most patients felt that, as laypeople, they didn’t have enough knowledge to actively participate in decisions about their care which created a sense of power imbalance and made shared decision-making (SDM) difficult which has been repetitively linked to poor physician-patient communication [30, 69, 77]. Patients with more medical experience felt empowered to engage actively in their care, viewing their physician as partners or consultants [77]. In order to encourage to ask questions, the use of a standard endorsement statement from physician which ensures that patients feel comfortable asking anything they want even if considered dumb or embarrassing can help [78]. SDM can be improved with the help of decision aids which are proven to provide more knowledge, greater satisfaction and making patients feel better informed [79–83]. While educational programs for physicians learning to focus on patient preferences in the context of SDM significantly improved the SDM [24], considering the complexity of communication, outcomes of communication skills training remain difficult to measure as other factors also play a role and training programs may require longer time [83–86]. Nevertheless, cancer centers should regularly assess patients on key indicators within dimensions of patient-centred care to determine if healthcare providers addressed their concerns, values, and preferences, offered support, and whether it alleviated their burdens [87]. 

A specific finding within the Tel Aviv cohort was the devastating impact of the armed conflict on the healthcare systems, patients and health care workers which resulted in significant psychological distress by disruption of medical care, lack of access to psychological help despite urgent need and disrupted or lost family support, a finding which was also reported from Ukraine calling for long term support [88–92]. Considering the challenge to cope with two simultaneous live threatening events (alloHSCT and war) and the challenges of access to psychological care, patients are at high risk for developing post-traumatic stress disorder by accumulation of known risk factors [93]. 

### Limitations

While the qualitative approach using semi-structured interviews including open questions offered the chance to capture issues not recognized yet, a few limitations need to be acknowledged. The sample consisted of patients from two centers only with differences in time since transplantation, educational level, diagnosis distribution, and cGVHD severity. These differences should be considered when interpreting cross-center similarities and differences in communication experiences. Moreover, the sample did not specifically target the impact of different cultural or ethnic backgrounds preventing any conclusions on topics like intercultural mismatches. Moreover, while the cohort include patients with a wide range regarding time after transplant the long-term survivors (> 2 years after transplantation) exclusively consisted of patients with cGVHD who required ongoing regular follow up visits limiting the experience to the latter long-term survivor population. In addition, the qualitative approach does not permit any meaningful analysis and associations of patient characteristics with specific challenges. Furthermore, the study’s findings may not be easily transferable to patients living alone as the majority of participants were married, which could distort the results because individuals living alone might face different challenges in communication and aftercare [53, 94]. Moreover, two researchers only conducted all interviews, which may have introduced potential researcher bias. Nevertheless, we attempted to maintain objectivity by discussing each step and finding with a senior team member to ensure the interpretations were as unbiased as possible. Efforts were made to minimize social desirability bias by having an interviewer who was not part of the transplant team.

## Conclusion

The results of this study reveal significant communication challenges faced by patients undergoing alloHSCT. The overwhelming influx of information during consultations, before and after transplantation, often leaves patients struggling to retain crucial details about their disease and treatment. Psychosocial distress, physical exhaustion, and cognitive impairments further exacerbate patients’ ability to engage with their healthcare providers. The complexity of conditions such as cGVHD, combined with patients’ physical and emotional fatigue, often hinders meaningful participation in physician-patient discussions, resulting in a power imbalance where patients feel compelled to trust their physician without full comprehension. Even though patients are convinced of their limited capacity to partake in their own medical decisions, the interviews showed that most of them think it is their responsibility rather than their physician to come forward with certain topics instead of being asked. The study also highlighted discrepancies in recognizing and addressing health issues, with many patients neglecting or minimizing symptoms they deemed less urgent or not related to recovery. The lack of adequate interdisciplinary communication, particularly during physician transitions, contributed to fragmented care and hindered a holistic understanding of patients’ needs. Additionally, patients frequently relied on relatives, nurses, or even personal search to fill informational gaps, underlining the importance of a functioning support network. Overall, the study calls for enhanced communication strategies for example with better training in communication skills for healthcare professionals, more use of decision aids and PRO measures, patient prompt lists and greater patient empowerment, and a more patient-centred approach to aftercare that addresses not only medical but also psychosocial concerns and individual needs.

## Supplementary Information

Supplementary Material 1.

## Data Availability

The detailed results of the framework analysis can be shared at reasonable request by the corresponding author. The content of the interviews cannot be shared due to data safety regulations.

## Abbreviations

cGVHD: ChronicGraft-versus-Host Disease
alloHSCT: Allogeneic hematopoietic stem cell transplantation (alloHSCT)
SDM: Shared decision making
IC: Informed consent
No: Number
yrs: Years
eICMs: eHealth-facilitated integrated care models
PROMs: Patient-reported outcome measures
