# Supplementary material for: Communication barriers in aftercare: a qualitative study of allogeneic stem cell transplant patients
Source: BMC Cancer. 2026 Jun 4;26:702. doi: 10.1186/s12885-026-16274-x (PMC13235129; doi:10.1186/s12885-026-16274-x)
Supplement: Supplementary file 1 — Supplementary Material 1. [file 12885_2026_16274_MOESM1_ESM.docx]

**Interview Guide**

Opening question: How would you describe your patient-physician conversations?

Communication of problems

- Now I would like to ask you to tell me which symptoms bothered you the most after the transplant.
  - If applicable: Which of these symptoms were the most stressful for you?
- Are there activities that you can no longer do but would like to do? If yes, which? Why doesn't it work anymore?
  - Were you able to communicate your symptoms/stress and were they noticed (especially if they worsened)? Has your physician asked about your symptoms (problems)? How was it discussed, was your problem responded to and if so, how? If not, why not?
  - Were there symptoms that you couldn't classify but the treatment team could (knowledge about GVHD, infections)?
  - Were individual symptoms of your GVHD or other complications not or only inadequately recorded or ignored (joints, skin, mouth, eyes, lungs, genitals?
  - How were fatigue symptoms (exhaustion not dependent on stress or time of day) discussed? – Did you tell the doctor, what was the reaction?
  - Did you experience neurocognitive limitations (forgetfulness, attention, confusion, driving)? If so, how was it discussed? What was your sleeping behaviour and was that recorded?
  - If you talked about it, did your physician react - if so, was the problem solved? If there was no response, why do you think there was no response (i.e., time constraints, no treatment options, other problems)?
  - How were your burdens surveyed? Can you remember whether problems were never or rarely discussed (i.e., mood (depression), various GVHD organ involvement)?
  - What are some reasons you haven't shared things? (i.e., no change expected, prefer to get through it alone)
  - Have you ever not addressed something because you didn't want to burden anyone and what was that?

Now I have a few questions in general about the discussions with your treatment team.

- Are there any problems/issues that you do not share with your treating doctor, even if he/she asks about them? If so, which ones, for example?
- Topics such as sexuality (genital GVHD, pain, erectile dysfunction), the desire to have children, different body feelings/feelings are often difficult to share, do you feel able to do so? How are/were these topics discussed? (This question was split in at least two questions in the interviews.)
- Sexuality not addressed, but expected to be addressed by the treatment team?
  - - Possible reasons: Did you not dare to speak up, other priorities/construction issues or did you want the impulse from the doctors?
- How was your mental health recorded? Was there any discussion about depressive moods (i.e., pressed, depressed mood; loss of interest and joylessness; lack of motivation and fatigue)?
- Have you been asked about fears?
- If such issues existed, who would you most likely share these issues with? (other physician (gender-specific issues), senior physician, psychologist, nurse, social service)
- Would it be easier for you to discuss sensitive topics if there wasn't a physician's assistant in the room?
- Would you like to be asked by your doctor about certain topics that you don't feel comfortable bringing up yourself?

Support situation in general

- Did you had or have questions about your medications and were you able to address them? (Prompt: new medication, old medications that may no longer be needed, discontinued medications that are missing without being communicated). How were these questions answered? (Prompt: from the doctor, understandable, etc.)
- Have the medications been explained? Do you understand why you need to take certain medications?
- Do you had or do you have any questions about the further progression of your illness? And if so, were you able to address these questions? How were these questions answered? (Prompt: from the doctor, understandable, etc.) How were the examinations/ check up´s that are necessary during the course explained to you (i.e., bone marrow aspirate: early detection of recurrent eye/derma: GVHD controls)?

Psychosocial situation

- How do you cope in everyday life? How are you cared for at home? If care is inadequate, does this impact treatment and do your providers know about it?
- Has it been recorded how you are doing (i.e., new physical and mental condition)? How often was this discussed?
- How do you cope with your limitations, what exactly are they and do you communicate them? Has this been noticed (especially deterioration)?
- Do you feel you receive adequate psychological care in the outpatient clinic? Was help offered?
- Is your social situation (sickness benefit, rehabilitation, pension) taken into account by the outpatient clinic and, if not, did you not communicate it or did the outpatient clinic not respond adequately?
- Has it been recorded how the relatives are coping with the illness?
- How did you find the support provided by the outpatient nursing staff (link-nurse) during and immediately after discharge from the hospital?
- Were you psychologically stressed (i.e., depression, fear) after discharge after the transplant - even if this had no direct connection to your illness - and did you or your physician report the stress and was there a response to them?

Offer of information

- After the transplant, do you think you were adequately informed about the possible consequences/complications of the treatment before the transplant? In your opinion, were side effects and consequences forgotten or explained incomprehensibly?
- Were you surprised by complications during the course of your illness (e.g. infection, GVHD) about which you wished you had been better informed beforehand? What complications came as a surprise to you?
- Have you sought information in any other way than through discussions with your treating doctors or bridge care? (Internet, books, exchange in online forums, with other patients) (Question is only asked, if the patient indicated alternative sources.)

Participatory decision making

- Did you feel included in the decision-making process regarding treatment (medications and diagnostics for complications, GVHD, infections), can you participate? Were your wishes taken into account or ignored?
- How was captured what is important to you? Were your needs recognized (e.g. after a proposed therapy)
- If not, on which topics would you have liked to have had more say?

Patient compliance and use of alternative treatment methods

- Were you able to take the medication as described in the medication plan? If no, why not and have you communicated this with your physician? This also includes medications that were still on the medication plan but have been already discontinued before. If you haven't communicated this, why? In case of discrepancies there physicians and nurses to whom it would have been easier to communicate to?
- Was there a situation where you had two different medication plans (from different disciplines) and how was that discussed?
- Does anyone else besides you have an overview of your medications/treatments?
- Have you tried alternative medication/treatments? (which and reasons)
- Have you informed the doctor about taking/using the alternative treatment methods and how did he/she react? If not, why didn't you communicate this or were you asked about it?

Positive events and stressors

- Can you describe your “worst” experience during the transplant with focus on the treatment in the outpatient clinic?
- What are the stressors of medical care for you and have you responded to them? (Are there situations (e.g. bone marrow aspirate) that you want to avoid and could this be made easier for you?)
- What things bother you most about visits to the outpatient clinic?
- How do you deal with stress factors? What would make it easier for you?
- What helps you in everyday life to better cope with the disease (GvHD) / life after the transplant?

Suggestions for improvement

- Do you have any other suggestions for communication between doctors and patients like you?
- Do you have any recommendations to improve aftercare?
- Do you have any suggestions regarding the organization/structure of aftercare?
- Ideal world: If there were no time problems, staff shortages and other factors, what would be the optimal doctor-patient consultation for you?

Sociodemographic questions:

- To better understand your situation, I have a few general questions:
- How old are you and how long ago did you have the transplant?
- Do you live alone or in a partnership/family?
- What job did you have before the transplant (school qualification)?
- Are you back to work or did the transplant lead to long-term restrictions? (This is only relevant from 6 months after the transplant.)
